# Sclerosing Osteomyelitis of Garré: A Clinico-Radiological Correlation

**DOI:** 10.7759/cureus.26866

**Published:** 2022-07-14

**Authors:** Fattah Rahiman Ghazali, Ahmad Hadif Zaidin Samsudin

**Affiliations:** 1 Radiology, School of Medical Sciences, Universiti Sains Malaysia, Kubang Kerian, MYS

**Keywords:** proliferative periostitis, non-suppurative sclerosing osteomyelitis, chronic osteomyelitis, ossifying periostitis, sclerosing osteomyelitis of garré

## Abstract

Sclerosing osteomyelitis of Garré is a rare and very specific type of chronic osteomyelitis that mainly affects children and young adults. To date, there is no clear etiology for the disease. Clinical findings and laboratory results are usually unremarkable with commonly negative blood and tissue cultures. Cortical thickening and periosteal reaction are common radiological findings. Biopsy often shows chronic non-specific inflammatory changes. It is a well-described entity in the dental literature, but to the best of our knowledge, there are no distinctive diagnostic criteria for long bones. We report a case of sclerosing osteomyelitis of Garré in a young lady involving the right tibia, for which the diagnosis was made based on clinico-radiological correlation.

## Introduction

Osteomyelitis (OM) is an inflammatory process of the bone that occurs in response to an infection, most commonly of bacterial origin. The pathophysiology of OM is well established in which there are five pathological phases: inflammation, suppuration, necrosis, new bone formation, and resolution [1]. Haematogenous spread is the main route of pathogenic spread. Exogenous spread such as direct inoculation due to trauma or surgical intervention is also known to cause bone infection. Ten clinical complications of acute osteomyelitis were described by Carl Garré in 1893. One of them is characterized by non-purulent sclerosis with thickening of the bone and elevation of the periosteum [2].

Sclerosing osteomyelitis of Garré is a rare and specific type of chronic osteomyelitis that mainly affects children and young adults. It is a well-described entity in the dental literature and the most commonly involved bone is the mandible [2,3]. Involvement of long bones such as femur and tibia has been reported [4]. A few terms have been used to describe this subtype, such as ossifying periostitis, chronic osteomyelitis with proliferative periostitis, and non-suppurative sclerosing osteomyelitis [3,4]. To date, there is still no clear etiology of the disease [5]. It is postulated that the chronic process is maintained by persistent low-grade infection or maintained even after the infection has been treated [5,6].

Males are more commonly affected. The patient usually seeks treatment after a long period of pain at the site of bone involvement as the onset is insidious. The symptoms also can recur at any time. Constitutional symptoms such as loss of appetite and loss of weight are usually absent. A distant secondary lesion can occur many years after the initial onset [4]. Physical examination is usually unremarkable. Localized findings such as swelling and tenderness over the affected bone may be found [6].

Laboratory results usually show mild elevation of inflammatory markers such as C-reactive protein (CRP) and erythrocyte sedimentation rate (ESR). Cultures from blood and tissue are usually negative. Radio imaging generally shows cortical thickening and periosteal reaction [5,7]. Isotope scans may show increased uptake at the involved site [7]. Biopsy results usually show chronic non-specific inflammatory changes [5-7].

Appropriate treatment for this disease is still unclear. Symptomatic relief is the aim of treatment in most cases. Analgesics, anti-inflammatory drugs, antibiotic therapy, and first-line surgical treatment have been reported to have a role in the management of patients in the past [5,7]. A new surgical technique which is bone resection followed by bone transport using a circular external fixator has shown promising result. However, this surgery should be reserved as the last option, especially after failed first-line treatment. In addition to that, more data are required for such a technique [6].

## Case presentation

Our patient is a 30-year-old lady who has complained of intermittent right leg pain for one year. The pain was aggravated by movement and cold weather while analgesics and rest only gave partial and temporary relief to her symptom. The application of a hot pack on her right leg somehow alleviated the pain. Otherwise, there were no swelling or skin changes, and she was able to walk normally and perform her daily chores. She denies prior history of trauma or fall, fever, constitutional symptoms such as poor appetite or weight loss, and a family history of malignancy. Physical examination was unremarkable. No abnormal gait, focal neurological deficit, local swelling, skin changes or tenderness. Blood parameters such as white cell count, hemoglobin, and platelet count were within the normal range except for raised inflammatory markers, specifically the CRP and ESR (20 mg/L and 92 mm/h, respectively). Other biochemical parameters revealed no significant abnormalities.

**Figure 1:**
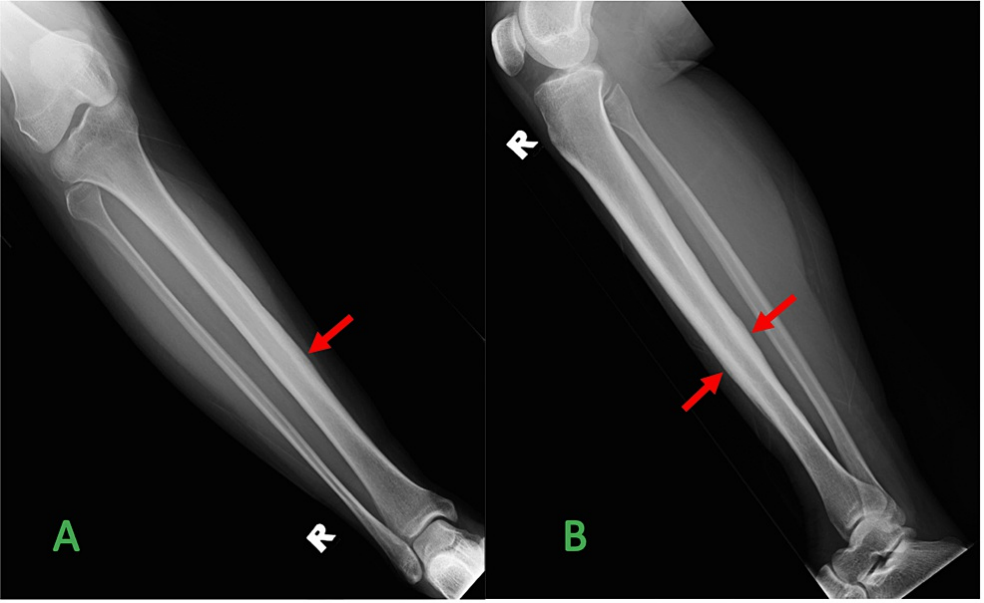
Radiograph of right tibia and fibula A: Frontal projection, B: Lateral projection shows cortical thickening of the shaft of the right tibia (arrows) with a normal cortical outline of the right fibula and the rest of the visualized bones

**Figure 2:**
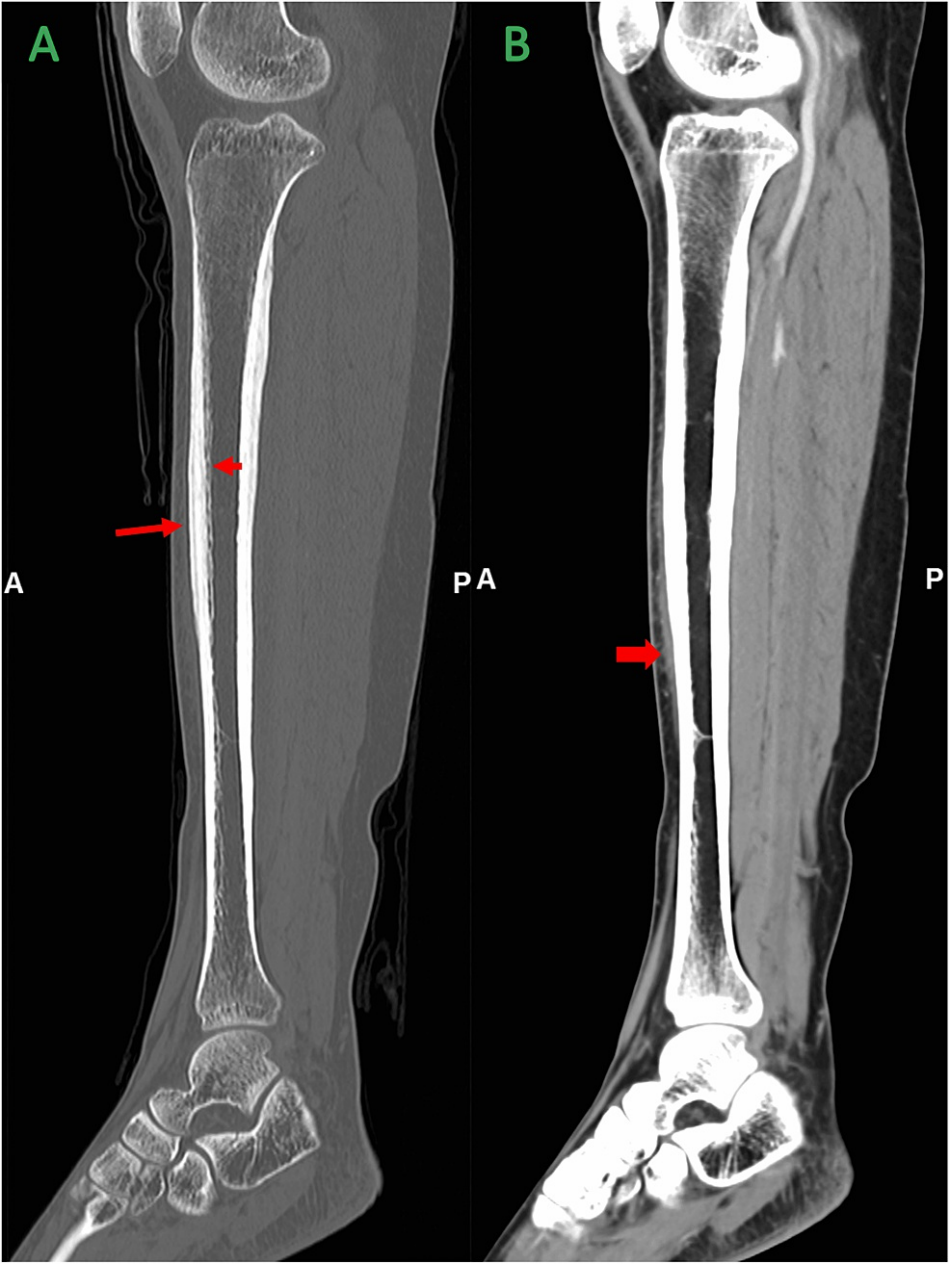
Sagittal CT images of right tibia A: Bone window, shows diaphyseal cortical thickening of the right tibia with solid, thick periosteal reaction (long arrow) and mild narrowing of marrow cavity (short arrow); B: Soft tissue window, shows associated subcutaneous tissue streakiness anteriorly (thick arrow) with no focal collection or intramuscular lesion

A plain radiograph of her right leg showed thickened cortex at the shaft of the tibia. The fibula was normal. No thickening of soft tissue was observed (Figure 1). Computed tomography (CT) scan revealed cortical thickening at the diaphysis of the right tibia with solid periosteal reaction. There was no focal lucency within the thickened cortex to suggest nidus. No fracture or aggressive feature such as bony destruction was noted (Figure 2, A). Adjacent soft tissue and overlying skin were normal (Figure 2, B). Magnetic resonance imaging (MRI) and radioisotope scan were not performed.

A core biopsy of the right tibia was performed, and the histopathological examination showed a mature bony cortex with no inflammation. There was no organism obtained or grown from the tissue culture. The patient was given analgesics by the orthopedic team and a short course of antibiotics during her hospitalization for the core biopsy procedure. She was also referred to the physiotherapist and underwent subsequent orthopedic follow-up. No further radiological imaging or surgical intervention was done. She was asymptomatic after two years of physiotherapy and orthopedic follow-up.

## Discussion

There is one subtype of OM that does not have the typical characteristics in terms of clinical presentation, physical examination, laboratory investigations, radiological findings, and histopathological examination. This subtype demonstrates mainly sclerotic changes rather than bone destruction as seen in classical OM. Different terminologies had been used for this subtype such as ossifying periostitis, chronic osteomyelitis with proliferative periostitis, and non-suppurative sclerosing osteomyelitis [2-4]. First described by Carl Garré in 1893 as one of the ten complications of OM, it is characterized by non-purulent sclerosis with thickening of the bone and elevation of the periosteum as a result of peripheral formation of a bone reaction due to irritation or mild infection [2,5].

Sclerosing osteomyelitis of Garré is a diagnosis of exclusion after more aggressive conditions have been ruled out and a thorough workup has been done. Differential diagnoses for sclerotic bone lesion may include osteoid osteoma, Ewing sarcoma, osteosarcoma and eosinophilic granuloma for neoplasm, and non-neoplastic condition such as Paget disease [5-7]. To the best of our knowledge, there are no distinctive diagnostic criteria discussed in the literature. Vannet et al. reported a case of sclerosing osteomyelitis of Garré in the femur based on the clinical and radiological features, with non-specific histological findings [4]. Song et al. described this rare disease as a confusing clinical diagnosis with difficult control of the symptom [7].

In our case, the diagnosis was made based on clinico-radiological correlation and after excluding other common differential diagnoses. Insidious, recurring pain on the affected site experienced by our patient is a common presentation of, although not specific to, the sclerosing osteomyelitis of Garré. Physical examination and laboratory investigations were generally normal and together with non-specific histopathological findings, made aggressive disease unlikely. Raised inflammatory markers, negative tissue culture and radiological findings in our patient are common features in sclerosing osteomyelitis of Garré.

The aim of treatment is targeted for symptomatic relief as well as eradication of the possible source of chronic inflammation. The management can be directed to first-line treatment with medications such as analgesics, anti-inflammatory drugs and antibiotics or by active surgical intervention or a combination of both. First generation cephalosporins such as cefazolin and cephalexin had been reported to be effective in symptom relief [7]. Clinical improvements by bisphosphonates had been shown in the case of diffuse sclerosing OM of the mandible [8]. Chang et al. reported a case of chronic osteomyelitis with proliferative periostitis in the lower jaw that became asymptomatic after administration of a non-steroidal anti-inflammatory drug [9]. Surgical intervention is indicated in cases that do not respond to the first line treatment [4,7]. Surgical approaches include intramedullary nailing and en bloc bone resection [4,10]. A newer surgical technique of bone resection and transport using a circular external fixator showed good functional result [6].

## Conclusions

A common presentation of sclerosing osteomyelitis of Garré is pain on the affected site. Unremarkable physical examination and biochemical results with negative cultures and non-specific histopathological features can contribute to inaccurate and delayed diagnosis. Cortical thickening or layering of the bone and periosteal reaction are common radiological findings, consistent with what we found in our case. Common and aggressive bone diseases need to be excluded. Prompt management usually results in clinical improvement.
